# The genomics of desmoplastic small round cell tumor reveals the deregulation of genes related to DNA damage response, epithelial–mesenchymal transition, and immune response

**DOI:** 10.1186/s40880-018-0339-3

**Published:** 2018-11-28

**Authors:** Andrea Devecchi, Loris De Cecco, Matteo Dugo, Donata Penso, Gianpaolo Dagrada, Silvia Brich, Silvia Stacchiotti, Marialuisa Sensi, Silvana Canevari, Silvana Pilotti

**Affiliations:** 1 Platform of Integrated Biology, Department of Applied Research and Technology Development, Fondazione IRCCS Istituto Nazionale dei Tumori di Milano, 2133 Milan, Italy; 2 Department of Diagnostic Pathology and Laboratory Medicine, Fondazione IRCCS Istituto Nazionale dei Tumori di Milano, 20133 Milan, Italy; 3 Department of Medical Oncology, Fondazione IRCCS Istituto Nazionale dei Tumori di Milano, 20133 Milan, Italy

**Keywords:** Desmoplastic small round cell tumor, Whole-exome sequencing, Somatic mutations, Copy number alterations, Chromosome imbalance, DNA damage response, Genomic stability, Mesenchymal–epithelial reverse transition/epithelial–mesenchymal transition, Immune response

## Abstract

**Background:**

Desmoplastic small round cell tumor (DSRCT) is a rare, aggressive, and poorly investigated simple sarcoma with a low frequency of genetic deregulation other than an Ewing sarcoma RNA binding protein 1 (*EWSR1*)-Wilm’s tumor suppressor (*WT1*) translocation. We used whole-exome sequencing to interrogate six consecutive pre-treated DSRCTs whose gene expression was previously investigated.

**Methods:**

DNA libraries were prepared from formalin-fixed, paraffin-embedded archival tissue specimens following the Agilent SureSelectXT2 target enrichment protocol and sequenced on Illumina NextSeq 500. Raw sequence data were aligned to the reference genome with Burrows–Wheeler Aligner algorithm. Somatic mutations and copy number alterations (CNAs) were identified using MuTect2 and EXCAVATOR2, respectively. Biological functions associated with altered genes were investigated through Ingenuity Pathway Analysis (IPA) software.

**Results:**

A total of 137 unique somatic mutations were identified: 133 mutated genes were case-specific, and 2 were mutated in two cases but in different positions. Among the 135 mutated genes, 27% were related to two biological categories: DNA damage-response (DDR) network that was also identified through IPA and mesenchymal–epithelial reverse transition (MErT)/epithelial–mesenchymal transition (EMT) already demonstrated to be relevant in DSRCT. The mutated genes in the DDR network were involved in various steps of transcription and particularly affected pre-mRNA. Half of these genes encoded RNA-binding proteins or DNA/RNA-binding proteins, which were recently recognized as a new class of DDR players. CNAs in genes/gene families, involved in MErT/EMT and DDR, were recurrent across patients and mostly segregated in the MErT/EMT category. In addition, recurrent gains of regions in chromosome 1 involving many MErT/EMT gene families and loss of one arm or the entire chromosome 6 affecting relevant immune-regulatory genes were recorded.

**Conclusions:**

The emerging picture is an extreme inter-tumor heterogeneity, characterized by the concurrent deregulation of the DDR and MErT/EMT dynamic and plastic programs that could favour genomic instability and explain the refractory DSRCT profile.

**Electronic supplementary material:**

The online version of this article (10.1186/s40880-018-0339-3) contains supplementary material, which is available to authorized users.

## Background

Desmoplastic small round cell tumor (DSRCT) is a very rare sarcoma arising from the abdominal/pelvic peritoneum that mainly occurs in young male adults and is included in simple sarcomas in the molecular sarcoma classification [1]. DSRCT is characterized by an ominous outcome as current aggressive polychemotherapies do not lead to a significant improvement in response and overall survival [2]; it is therefore included in the “most wanted” list of sarcomas requiring new agents [3].

The hallmark of DSRCT is the specific t(1;22)(p13;q12) translocation that leads to the fusion of the Ewing sarcoma RNA binding protein 1 (*EWSR1*) and Wilm’s tumor suppressor (*WT1*) genes. Most of other reported molecular alterations occur in single cases. The MET proto-oncogene, receptor tyrosine kinase (*c*-*MET)* N375S mutation (the most frequently encountered in lung carcinoma) and two mutations in phosphatidylinositol-4,5-bisphosphate 3-kinase catalytic subunit alpha (*PI3KCA*) have been described in 2 of 10 pre-treated DSRCTs interrogated using a single-gene polymerase chain reaction (PCR)-based assay [4]. Evidence indicating the involvement of epigenetic regulators has been reported in two cases: the first, investigated using immunohistochemistry (IHC), showed the complete loss of SWI/SNF-related, matrix-associated, actin-dependent regulator of chromatin, subfamily b, member 1 (SMARCB1/INI) protein [5], and the other, investigated using targeted next-generation sequencing (NGS), showed a mutation in AT-rich interaction domain 1A (*ARID1A*) gene corresponding to a variant of unknown significance [6]. More recently, whole-exome sequencing (WES) analysis of a thoroughly investigated, pre-treated case revealed the presence of 15 acquired somatic mutations [7], 7 of which were regulated by the same lymphoid enhancer binding factor 1 (LEF1) transcription factor which, in addition to being involved in the Wnt/beta-catenin signalling pathway, is a facilitator of the epithelial–mesenchymal transition (EMT) [8]. Finally, amplification of aurora kinase B (*AURKB*) and MCL1, BCL2 family apoptosis regulator 1 (*MCL1*) has been reported in one case of DSRCT interrogated using targeted NGS [6], and gains in chromosome 5 and 18 and loss at 13q have been detected in one case interrogated using WES and comparative genomic hybridization [7]. In the present study, we used WES to investigate six cases of DSRCT known to carry the specific *EWSR1*-*WT1* translocation and was previously investigated using gene expression profiling complemented by immunophenotyping, microRNA (miRNA) in situ hybridisation (ISH), and a cancer stem cell array analysis [9].

## Materials and methods

### Patients and study design

Seven consecutive cases of primary DSRCT that were surgically removed after multi-drug chemotherapy between 2000 and 2016 were analyzed. The presence of the *EWSR1*-*WT1* translocation was detected using fluorescent in situ hybridisation coupled with immunolabeling restricted to WT-C19 [9]. The clinical data (including follow-up data when available) were obtained from the patients’ records and were previously described [9]. The study was approved by the Independent Ethics Committee of the Fondazione IRCCS Istituto Nazionale dei Tumori di Milano. All patients gave their written consent to donating the tissue remaining after diagnostic procedures. One of the seven cases was excluded from WES analysis due to insufficient DNA quantity.

### DNA library preparation

The WES analysis was made using materials dissected from ten 7-μm methylene blue-stained sections of non-necrotic tissue representative of tumoral areas, which were paired with the corresponding adjacent normal tissue in formalin-fixed, paraffin-embedded (FFPE) archival tissue specimens. DNA was extracted using a GeneRead DNA FFPE kit (Qiagen, Germantown, MD, USA) and quantified using a Qubit Fluorometer (ThermoFisher, Waltham, MA, USA). Shearing of 500 ng of DNA was carried out in 50 μl of 1× TE buffer using a Covaris M220 equipped with microTUBE AFA fibre tubes and SonoLab 7.2.0.64 software (Covaris Inc., Woburn, MA, USA). The resulting size distribution, which was about 160 bp in all cases, was checked using a TapeStation 4200 (Agilent, Santa Clara, CA, USA). The libraries were prepared using SureSelectXT2 Reagent kit (Agilent) in accordance with the manufacturer’s instructions and included the end-repair of fragmented DNA, A-tailing, adapter ligation and amplification, with purifications being carried out between each step using Agencourt AMPure XP beads (Agilent). The yield of the constructed libraries was estimated using a Qubit dsDNA HS kit (Qiagen). Exomes were captured using SureSelectXT2 Human All Exon probes (Agilent). Hybridisation of the pooled libraries with the capture probes, the removal of any non-hybridised library molecules, and PCR amplifications were carried out in accordance with Agilent SureSelectXT2 instructions. The captured libraries were then sequenced on a NextSeq 500 sequencer (Illumina, San Diego, CA, USA), with sample dilution, flow cell loading, and sequencing being carried in accordance with Illumina specifications.

### Sequencing data analysis

The workflow of the analyses is reported in Additional file 1: Figure S1. Raw unmapped reads of the tumor and normal sample were aligned to the human reference genome (hg19 build) using the Burrows–Wheeler Aligner (BWA)-enrichment application v2.1.0 of Illumina BaseSpace [10]. After alignment, we removed, from sorted and indexed compressed binary version of Sequence Alignment Map (BAM) files, unmapped reads with samtools v1.3.1 [11] and duplicate reads with Picard software v1.79 (http://broadinstitute.github.io/picard/). We then used Genome Analysis Toolkit (GATK) v3.7 [12] to perform left alignment of small insertions and deletions (indels), indel realignment, and base quality score recalibration. Concordance and cross sample contamination was assessed using the computational method Conpair which detects cross sample contamination among tumor-normal pairs at levels as low as 0.1%, even in presence of copy number changes [13]. Somatic variant calling was performed on tumor-normal pairs using MuTect2 v3.7 [14]. The criteria to remove possible false positive mutations induced by FFPE or oxidative DNA damage [15, 16] are reported in the legend of Additional file 1: Figure S1. Oncotator [17] was used to annotate point mutations and indels with functional data and functional consequences. A mutation was defined deleterious if at least one algorithm among MutationAssessor [18], MutationTaster [19], and SIFT [20] assigned a deleterious effect on the function of a protein affected by somatic mutation.

Somatic CNAs from WES data were identified using EXCAVATOR2 [21]. The mutational signatures designated in Catalogue Of Somatic Mutations In Cancer (COSMIC; https://cancer.sanger.ac.uk/cosmic) were analyzed on the basis of the SNVs and their sequence context, considering the immediately flanking 5′ and 3′ nucleotides using the R package deconstructSigs [22]. This analysis was performed aggregating mutations from all six cases.

### Functional pathway and manual curation analyses

Mutations were analyzed for presence in COSMIC and in the Cancer Census collection of genes which contain mutations that have been causally implicated in cancer (downloaded on 18 February 2018 from http://cancer.sanger.ac.uk/census).

Molecular and cellular function analyses of the mutated genes and of the genes present in the aberrant chromosomal regions were carried out using Ingenuity^®^ Pathway Analysis (IPA^®^, Qiagen; Bioinformatics, Redwood City, CA, USA; http://www.qiagen.com/ingenuity). Manual curation of selected mutated/deregulated genes was done exploiting previous knowledge and the following websites: http://www.ncbi.nlm.nih.gov/pubmed; http://cancer.sanger.ac.uk/cosmic; and https://cancergenome.nih.gov.

### Statistical analyses

A right-tailed Fisher’s exact test, corrected for multiple testing by the Benjamini–Hochberg false discovery rate (FDR), was used to calculate *P* values for IPA over-representation analysis. An FDR < 0.05 was considered to select significantly enriched pathways. Pearson’s correlation coefficient was used to compute correlation between the total number of mutations and chromosome length. The statistical significance of mutational signatures was calculated according to the R package deconstructSigs [22]. Mutational signatures with > 10% weight (> 0.1) were considered to have substantial contribution to the overall mutational landscape.

## Results

### Quality control of sequence data

We performed WES in six cases of DSRCT that, on the basis of previously published results of transcriptome profiling, microRNA (miRNA) in situ hybridisation (ISH), and IHC assays, were divided into three groups that recapitulated the traits of mesenchymal–epithelial reverse transition (MErT), hybrid/partial EMT, and EMT [9]. After alignment of raw reads, we evaluated the quality of data in terms of coverage and sample purity. Analysis with Conpair showed that each tumor-normal pair was correctly matched: a sample concordance > 99% and negligible levels of cross-sample contamination among tumor-normal pairs were observed (Additional file 1: Figure S2a, b). The mean coverage, after removal of duplicate reads, ranged from 109.4× to 123.9× for tumor samples and from 52.3× to 71.7× for normal samples (Additional file 1: Figure S2c). We also estimated the percentage of target bases covered at least 50× that ranged from 71.4% to 88.8% for tumor samples and from 46.0% to 71.9% for normal samples (Additional file 1: Figure S2d).

After variant calling and filtering to remove possible artefacts and false positive variants (see criteria in Additional file 1: Figure S1), we identified 137 somatic mutations affecting 135 genes (listed in Additional file 2: Table S1).

### Mutation spectrum across the cases

**Fig. 1:**
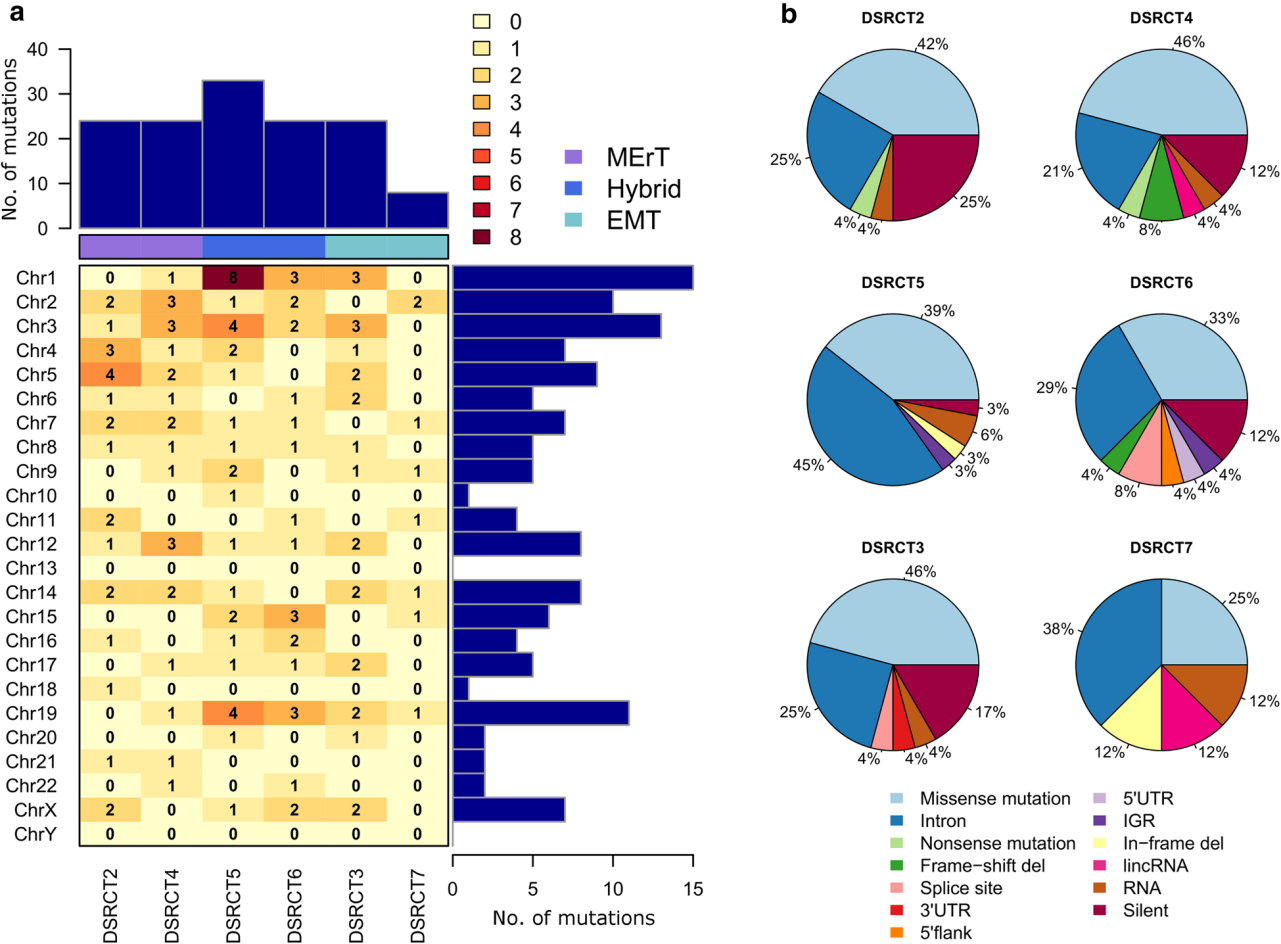
Mutation spectrum of desmoplastic small round cell tumor (DSRCT). **a** Color-coded heatmap reporting the number of observed somatic mutations identified in each chromosome for each case. The total number of mutations for single case and their chromosome locations are reported in the vertical and horizontal bar plots, respectively. The cases are classified into three groups (see coloured top horizontal bar). **b** Distribution of somatic variant types for each case. MErT, mesenchymal–epithelial reverse transition; hybrid, hybrid/partial epithelial–mesenchymal transition; EMT, epithelial–mesenchymal transition

Each case was characterized by a sizeable number of mutations: from 8 to 33 mutations per case (mean, 23) (Fig. 1a). The number of mutations for each case according to chromosomal location indicated a non-preferential pattern of distribution (Fig. 1a) and a positive correlation between the total number of mutations/chromosome and chromosome length (Pearson’s *r* = 0.67). A consistent proportion of mutations were categorized as missense or intronic, followed by silent mutations (Fig. 1b).

**Fig. 2:**
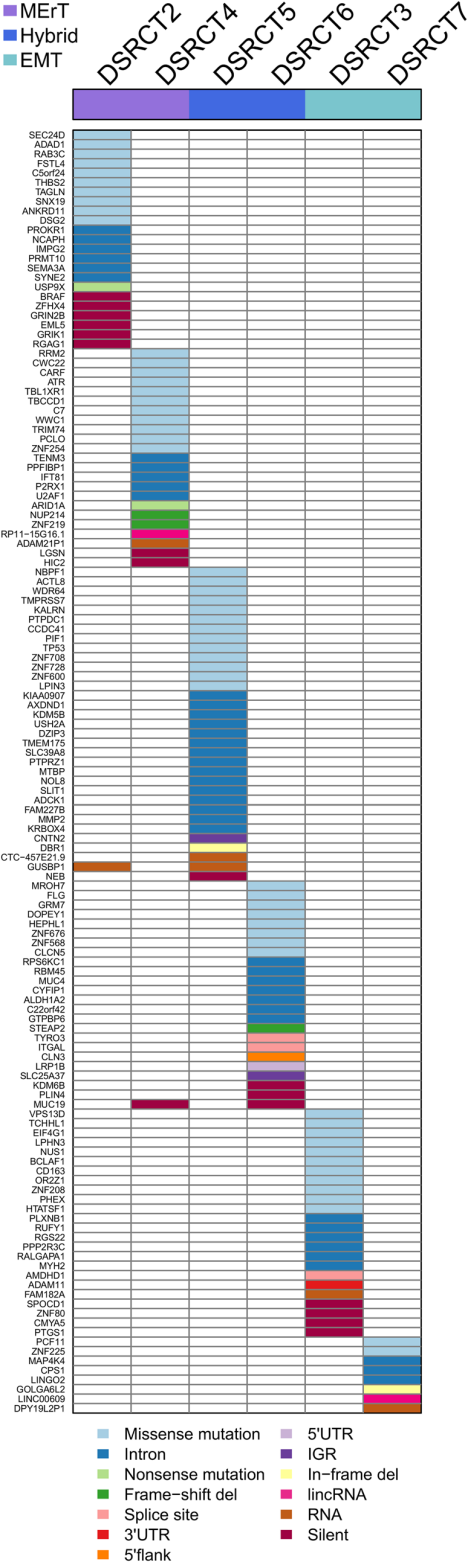
Inter-patient heterogeneity of mutated genes in DSRCTs. Heatmap, coloured according to the variant type, representing the 135 mutated genes across six cases of DSRCT. The cases are classified into three groups (see coloured top horizontal bar). MErT, mesenchymal–epithelial reverse transition; hybrid, hybrid/partial epithelial–mesenchymal transition; EMT, epithelial–mesenchymal transition. For the entire name of the genes, reported as gene ID, see Additional file 2: Table S1

Figure 2 shows the inter-patient heterogeneity of mutated genes across the six DSRCT cases. Among them, 135 were case-specific and only two genes, mucin 19 (*MUC19*) and glucuronidase, beta pseudogene (*GUSBP1*), were found mutated in two cases but in different positions.

### Comparison between the mutational profile and COSMIC mutational signatures

**Fig. 3:**
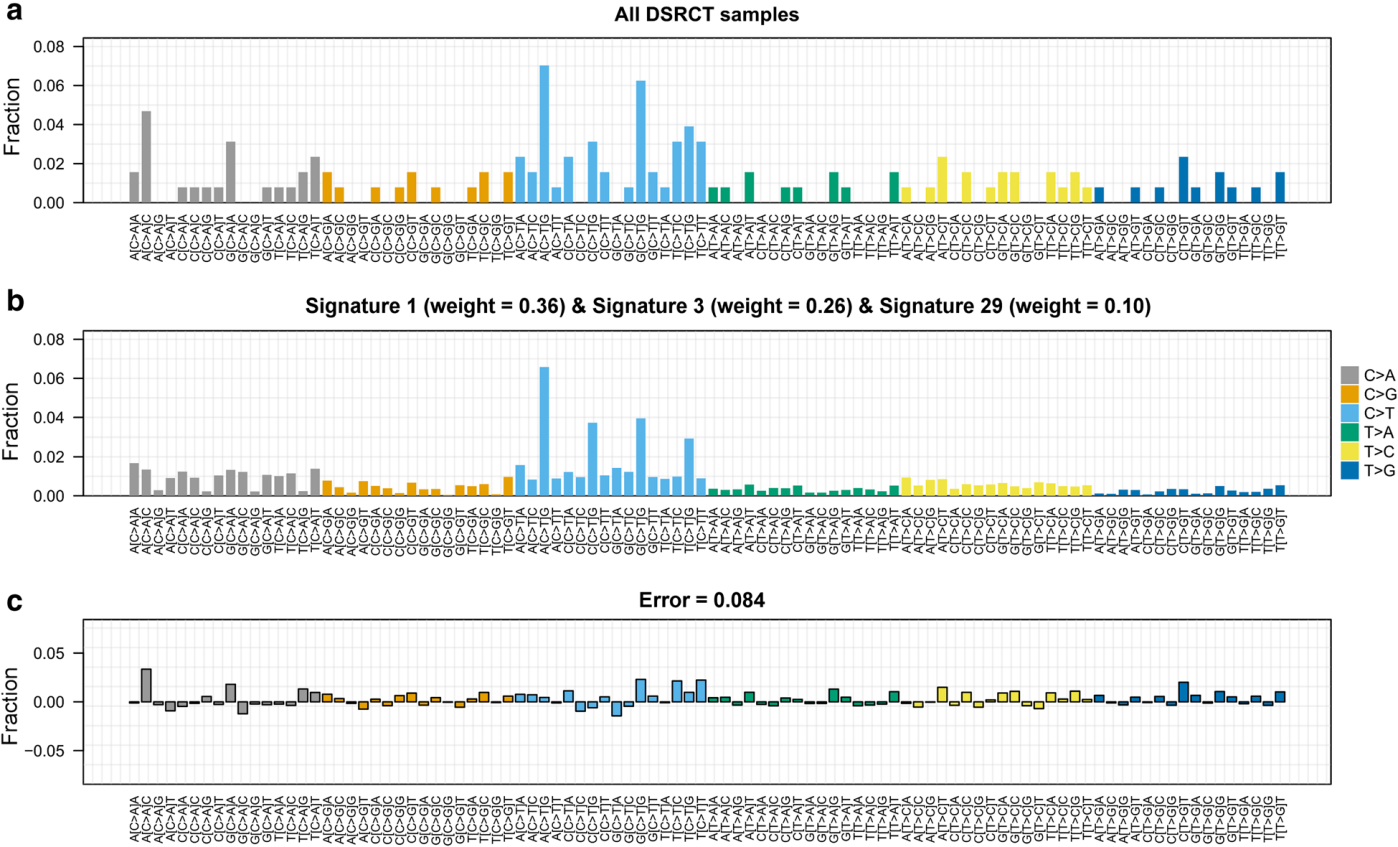
Comparison between the DSRCT mutational profile and mutational signatures from Catalogue Of Somatic Mutations In Cancer (COSMIC). **a** Observed mutational profile of the aggregated list of the 137 somatic mutations categorized according to the 96 possible tri-nucleotide variations. The fraction of mutations found in each trinucleotide context is displayed. **b** Reconstruction of the observed DSRCT profile according to the 30 COSMIC mutational signatures; known COSMIC signatures which gave the major contribution (weights) are reported at the top of the panel. **c** Differences between the observed and the reconstructed mutational profiles; the mean error is reported at the top of the panel

Before surgery, all patients had received 3–6 cycles of multi-drug chemotherapy including one or two alkylating agents [9]. To evaluate whether the identified mutations may have been due to the dominant effect of chemotherapy, we tested 30 mutational signatures from COSMIC in the aggregated list of our 137 somatic mutations. The comparison between the observed (Fig. 3a) versus the reconstructed (Fig. 3b) mutational profiles with an irrelevant error (Fig. 3c) indicated that the DSRCT profile essentially derived from the contribution of three mutational signatures denominated in COSMC as 1, 3 and 29 (respective weights: 0.36, 0.26, and 0.10); the mutational signature associated with treatment with alkylating agents (signature 11, weight = 0) did not contributed to mutational profile.

### Mutated genes, associated pathways, and manual curation

**Fig. 4:**
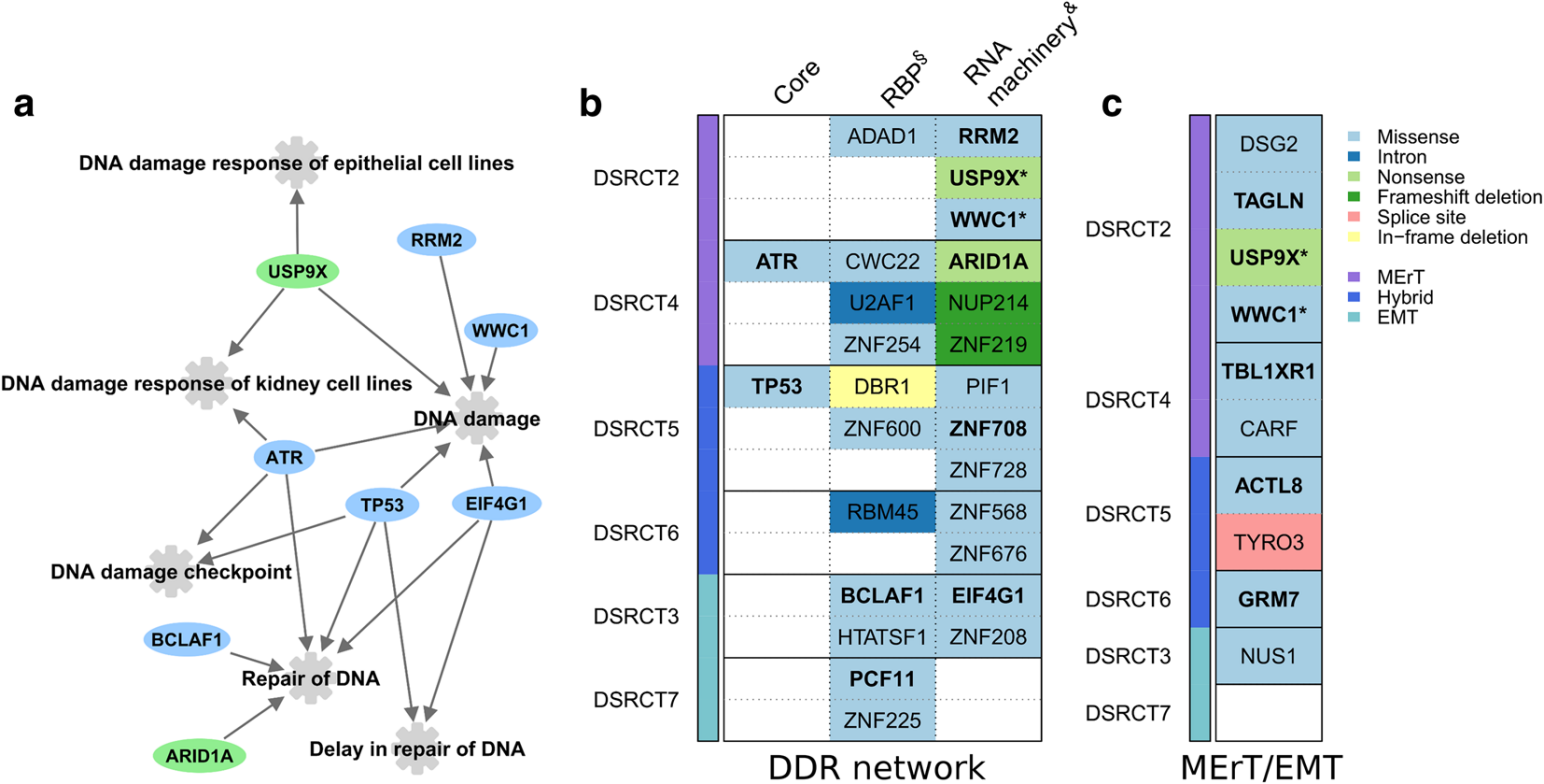
Pathways and genes mutated in DSRCT. **a** Network representing the six biological pathways containing protein-encoding mutated genes from our list significantly enriched according to Ingenuity Pathway Analysis (IPA^®^, Qiagen; Bioinformatics, Redwood City, CA, USA; http://www.qiagen.com/ingenuity). **b** Heatmap reporting the 26 mutated genes across the six cases by the three DNA damage response (DDR) network subsets: core, RNA-binding proteins (RBPs), and RNA machinery (either directly or indirectly related). **c** Heatmap representing the distribution of the 10 mutated genes belonging to the MErT/EMT process across the six cases. The cases are classified into three groups (see coloured vertical bars on the left). Genes with a deleterious mutation are highlighted in bold. Genes belonging to both DDR network and MErT/EMT are marked by an asterisk. Genes are coloured according to the type of mutation. MErT, mesenchymal–epithelial reverse transition; hybrid, hybrid/partial epithelial–mesenchymal transition; EMT, epithelial–mesenchymal transition. Gene names: *ADAD1*, adenosine deaminase domain containing 1; *RRM2*, ribonucleotide reductase regulatory subunit M2; *USP9X*, ubiquitin specific peptidase 9 X-linked; *WWC1*, WW and C2 domain containing 1, *WWC1*; *ATR*, ATR serine/threonine kinase; *CW22*, CWC22 spliceosome associated protein homolog; *ARID1A*, AT-rich interaction domain 1A; *U2AF1*, U2 small nuclear RNA auxiliary factor; *NUP214*, xx; *ZNF254*, zinc finger 254; *ZNF219*, zinc finger 219; TP53, Tumor protein p53; *DBR1*, debranching RNA lariats 1; *PIF1,* PIF1 5′-to-3′ DNA helicase; *ZNF600*, zinc finger 600; *ZNF708*, zinc finger 708; *RBM45*, xx; *ZNF568*, zinc finger 568; *ZNF676*, zinc finger 676; *BCLAF1*, BCL2 associated transcription factor 1; *EIF4G1*, eukaryotic translation initiation factor 4 gamma 1; *HTATSF1*, HIV-1 Tat specific factor 1; *ZNF208*, zinc finger 208; *PCF11*, xx; *ZNF225*, zinc finger 225; *DSG2*, xx; *TAGLN*, transgelin; *TBL1XR1*, transducing beta like 1 X-linked receptor 1; *CARF*, calcium responsive transcription factor; *ACTL8*, actin like 8; *TYRO3*, TYRO3 protein tyrosine kinase; *GRM7*, glutamate metabotropic receptor 7; *NUS1*, NUS1, dehydrodolichyl diphosphate synthase subunit

Functional analysis by using IPA indicated that the 135 genes affected by damaging mutations in DSRCTs caused enrichment in the following biological pathways: DNA damage response (DDR) of kidney cell lines, DNA damage, delay in repair of DNA, repair of DNA, DNA damage checkpoint, and DDR of epithelial cell lines (all *P* < 0.001) (Fig. 4a).

Manual curation of the list of mutated genes was based on the recent evidence that genes encoding RNA-binding proteins (RBPs) and DNA/RNA-binding proteins (DRBPs) as well as other genes related to RNA machinery are strictly connected to DDR [23] and on our previous data [9]. According to this section, we found that 27% of the 135 mutated genes belonged to the DDR network (Fig. 4b) or to MErT/EMT process (Fig. 4c).

Categorization in these two biological processes showed that each case harboured mutation in at least one gene for each category. Details regarding each gene hereafter described, such as full name, a summary from NCBI Entrez Gene database (http://www.ncbi.nlm.nih.gov/gene) and PubMed (http://www.ncbi.nlm.nih.gov/pubmed) and their presence in COSMIC and in the Cancer Census are reported in Additional file 2: Table S2.

### Mutations in the DDR network

The DDR network was affected by 26 mutated genes that can be grouped in three subsets: core genes, genes encoding RBPs, and other genes related to RNA machinery.

#### Core genes

A damaging missense mutation in ATR serine/threonine kinase (*ATR*), one of the two core genes of DDR (the other is serine/threonine kinase, *ATM*), was found in DSRCT4 of the MErT group. The function abrogation of this gene could allow tumors to escape checkpoint control and could lead to uncontrolled replication (see also references in Additional file 2: Table S2).

Tumor protein p53 (*TP53*), even not a canonical core gene of DDR, being a master regulator of transcription and clearly associated with DDR, was included in the subset of core genes. The mutation here found in DSRCT5 of the hybrid EMT group was a deleterious missense mutation that was localized in the canonical hotspot disabling the encoded protein. The allele frequency of this mutation was low (12.9%, Additional file 2: Table S1), but this value may be considered in agreement with the strong p53 nuclear immunostaining with a patchwork pattern in half of the tumor cells (data not shown).

#### Genes encoding RBPs

All six cases had at least one mutation affecting RBP genes. Among the 12 identified mutations, 2 were intronic, 1 was an in-frame deletion, and 6 were missense (Fig. 4b), being mutations in BCL2-associated transcription factor 1 (*BCLAF1*) and cleavage and polyadenylation factor subunit (*PCF11*) genes predicted to be damaging.

Beside *BCLAF1* and *PCF11*, three other mutated genes [CWC22 spliceosome-associated protein homolog (*CWC22*), debranching RNA lariats 1 (*DBR1*), and adenosine deaminase domain containing 1 (*ADAD1*)] are involved in pre-mRNA production, defective DNA repair, miRNA and snoRNA regulation with prevalence in the early step of spliceosome editing. In particular, *ADAD1* function has not yet been deciphered but, on the basis of the hypothesis that the adenosine deaminase acting on RNA (ADAR) enzyme family controls RNA editing, an alteration in one of its members may affect a wide range of RNA processing activities [24, 25]. The function of HIV-1 Tat specific factor 1 (*HTATSF*) gene is also largely unknown, but its mutation has been reported to parallel the decreased expression of many genes [26].

Zinc finger proteins (ZNFs) belong to a number of different structural families. Three out of *ZNF* mutated genes (*ZNF254, ZNF600*, and *ZNF225*) were annotated among the RBP subset because they belong to the class of Cys2-His2 (C2H2) ZNFs and thus are expected to act consistently [27].

Finally, two additional genes, U2 small nuclear RNA auxiliary factor (*U2AF1*) and RNA binding motif protein 45 (*RBM45*), were found mutated at an intron site, and such mutations might have a negative impact on translational efficiency, as reported [28, 29].

#### Other genes related to RNA machinery

This group included nine genes with a missense mutation, four of which with damaging effect, and two genes with a damaging nonsense mutation (Fig. 4b).

The genes affected by a damaging mutation were ribonucleotide reductase regulatory subunit M2 (*RRM2*), whose deregulation was reported to impair a key step in DNA synthesis; *ARID1A*, whose wild type-encoded protein acts as an epigenetic regulator; eukaryotic translation initiation factor 4 gamma 1 (*EIF4G1*), whose deregulation could negatively affect the correct mRNA circularization and/or translation activity downstream of serine/threonine-protein kinase mTOR; *ZNF708*, that, like RBPs, is reported to act as interaction module with DNA, RNA, proteins, and other molecules and whose deregulation could affect gene transcription, translation, and mRNA trafficking [30].

The remaining two genes [ubiquitin specific peptidase 9 X-linked (*USP9X*) and WW and C2 domain containing 1 (*WWC1*)], harbouring damaging mutations, are described in the following subsection since they are also involved in MErT/EMT.

### Mutations in MErT/EMT genes

Eight mutated genes are reported to be directly involved in MErT/EMT. Four genes [transgelin (*TAGLN1*), ubiquitin specific peptidase 9, X chromosome (*USP9X*), WW and C2 domain containing 1 (*WWC1*), and transducing beta like 1 X-linked receptor 1 (*TBL1XR1*)], harbouring missense damaging mutations, were found in the MErT group, strongly supporting the hypothesis that such mutations induced changes consistent with an epithelial-like phenotype. In particular, the two mutated genes *(USP9X* and *WWC1*) present in both biological categories were found in the DSRCT2 case, paradigm of MErT being, according to IHC, the most enriched in epithelial-related molecules [9].

Two damaging mutations were also found in the actin like 8 (*ACTL8*) and glutamate metabotropic receptor 7 (*GRM7*) genes, with each one recorded in one of the cases of the Hybrid EMT group. Interestingly, aberrant expression of *ACTL8*, a cancer/testis antigen gene, is reported to associate with stem cell-like enrichment and an EMT signature [31], both of which are characteristics of DSRCT. As *GRM7*, it has been proposed that its silencing may provide a further mechanism that regulates MErT/EMT by inhibiting TGFbeta/SNAIL via AMPK activation.

A non-damaging missense mutation was detected in dehydrodolichyl diphosphate synthase subunit (*NUS1*) gene in a case belonging to the EMT group. High expression of this gene is reported to associate with EMT, and its silencing with MErT. Furthermore, missense non-damaging mutations were observed in two genes, deasmoglein2 (*DSG2*) and calcium-responsive transcription factor (*CARF*), whose involvement in MErT is indirectly suggested: for *DSG2*, by its belonging to a cadherin cell adhesion molecule superfamily; for *CARF*, by its contribution to WNT signalling activation.

Regarding the non-damaging splice-site mutation in TYRO3 protein tyrosine kinase (*TYRO3*), the aberrant SNAIL-mediated expression of *TYRO3* is reported to be associated with EMT.

### CNA landscape of DSRCT

**Fig. 5:**
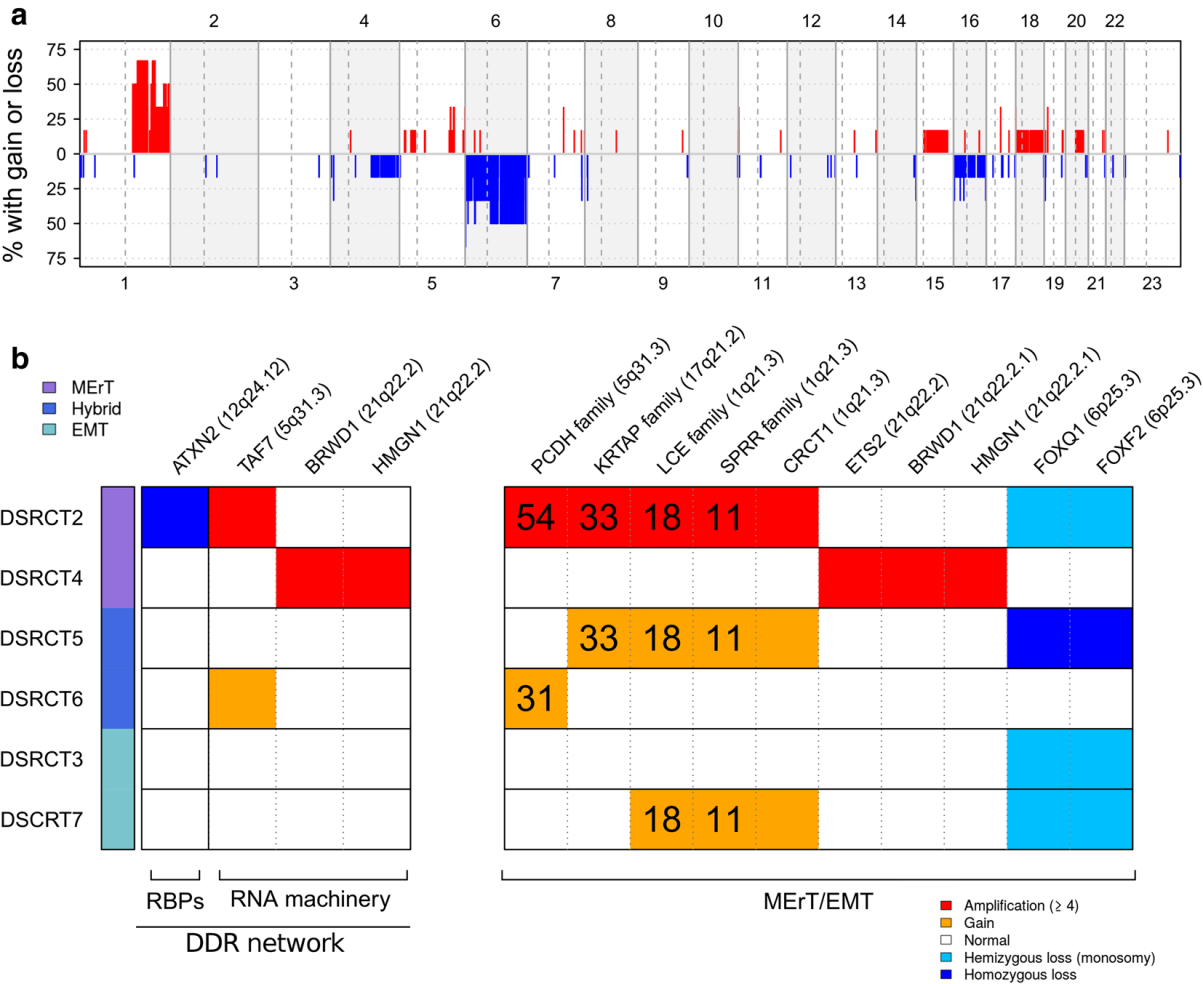
Copy number alteration (CNA) landscape of DSRCTs. **a** Genome-wide frequency of CNAs according to EXCAVATOR2 in the six cases of DSRCT; copy number gains and losses are reported in red and blue, respectively. **b** Gains and losses across the six cases for DDR network (left part) and MErT/EMT (right part). CNA events are colored according to EXCAVATOR2 copy number call. On the top are the genes belonging to these CNAs and their corresponding cytoband. Numbers on the box represent the number of genes involved. The cases are classified into three groups (see coloured vertical bars on the left). MErT, mesenchymal–epithelial reverse transition; hybrid, hybrid/partial epithelial–mesenchymal transition; EMT, epithelial–mesenchymal transition. Gene names: *ATXN2*, ataxin 2; *TAF7*, TATA box-binding protein-associated factor; *BRWD1*, bromodomain and WD repeat domain containing 1; *HMGN1*, high mobility group nucleosome-binding domain 1; *PCDH*, protocadherin; *KRTAP*, keratin-associated protein; *LCE*, late cornified envelope; *SPRR*, small proline-rich protein; *CRCT1*, cysteine-rich C-terminal 1; *ETS2*, ETS proto-oncogene 2; *FOXQ1*, forkhead box Q1; *FOXF2*, forkhead box F2

The pattern of somatic CNAs showed several regions amplified or lost in a case-specific fashion in addition to large amplifications of the long arm of chromosome 1 recurring in more than 50% of cases and complete or partial loss of chromosome 6 present in 50% of cases (Fig. 5a). All CNA events, as details of category, chromosome location, gene in the region and cytoband, are reported in Additional file 2: Table S3.

### CNAs in DDR and MErT/EMT genes

Focusing on gain and loss of copy number (Additional file 2: Table S4), we found that a sizable number of CNA-affected genes were recurrent across patients and affected both DDR and MErt/EMT; details for each of the genes cited below are reported in Additional file 2: Table S2.

Among the DDR category, the following genes displayed gain/amplification (Fig. 5b, left part): TATA box-binding protein-associated factor (*TAF7*), amplified in two cases, and reported to be involved in very early steps of transcription; high mobility group nucleosome-binding domain 1 (*HMGN1*) and bromodomain and WD repeat domain containing 1 (*BRWD1*), playing a role in chromatin structure and remodelling. A homozygous deletion of ataxin 2 (*ATXN2*), codifying for a RBP, was observed; it has been reported that silencing of *ATXN2* gene, which is known to affect neurodegenerative diseases, led to disturbance in RNA transcription.

Overall CNAs mostly segregated in the MErT/EMT category (Fig. 5b, right part). In particular, in DSRCT2 we found high amplification of many genes associated to squamous or terminal squamous differentiation, late cornified envelope (*LCE,* 18 genes) gene family, small proline-rich protein family (*SPRR*, 11 genes), and cysteine rich C-terminal 1 (*CRCT1*), to sulfur hair keratin (keratin-associated protein, *KRTAP*, 33 genes) family, and brain-specific cadherin-like adhesion molecules (protocadherin, *PCDH*, 54 genes). Many of these genes showed recurrent gains (three copies) in DSRCT5 and 6, and genes of the *PCDH* family were also present in DSRCT6.

ETS proto-oncogene 2, transcription factor (*ETS2*) amplification was present in DSRCT4 of the MErT group. It has been reported, at preclinical level, that *ETS2* is a specific master factor, able to promote hybrid EMT by directly binding (and thus preventing) miRNA-200 transcription.

*FOXQ1* and *FOXF2* genes on chromosome 6 were present in homozygous deletion in DSRTC5 of the hybrid EMT group and in heterozygous deletion in DSRTC2, 3, and 7.

It has been demonstrated that *FOXQ1* is a critical mediator of EMT, and, in our cases, the null (DSRCT5) or attenuated (DSRCT2, 3, and 7) profiles dictated by the defective status of the gene were in line with both the gain/amplification of the epithelial-related genes found here (DSRCT2, 5, and 7) and the previously reported expression of E-cadherin/miRNA-200 module in DSRCT2 and 5 [9].

In vitro *FOXF2* loss acts as an EMT-suppressing transcription factor whose deficiency induces EMT through twist family bHLH transcription factor 1 (TWIST) up-regulation, a finding consistent with zinc finger E-box binding homeobox 1 (ZEB1) expression observed in DSRCT3, 5, and 7 [9].

### Imbalances of chromosomes 1 and 6

**Fig. 6:**
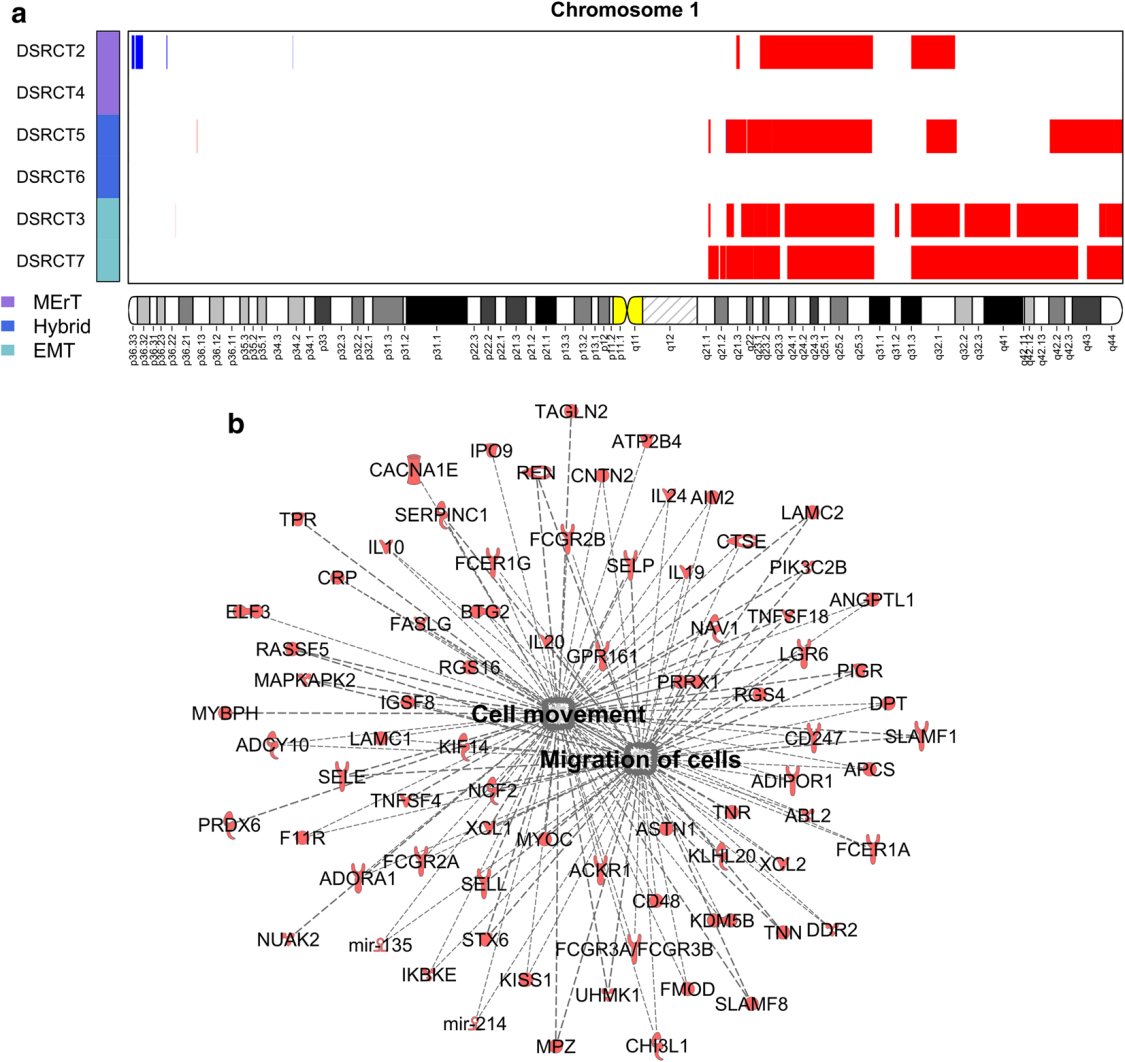
Chromosome 1 gains in DSRCTs. **a** Plot representing genomic location of amplified segments of chromosome 1 in the six DSRCT cases. Recurrent gains were identified in four cases (DSRCT2, 3, 5, and 7). The cases are classified into three groups (see coloured vertical bars on the left). **b** Network showing genes mapping to chromosome 1 and associated to the functional categories by Ingenuity Pathway Analysis (IPA^®^, Qiagen; Bioinformatics, Redwood City, CA, USA) and significantly enriched in the list of 410 genes commonly amplified in the four cases. The most enriched categories were “Cell movement” and “Cell migration” (both *P* < 0.001). See also Additional file 2: Table S6 for gene overlapping between the two pathways. For the entire name of the genes, reported as gene ID, see Additional file 2: Table S5

**Fig. 7:**
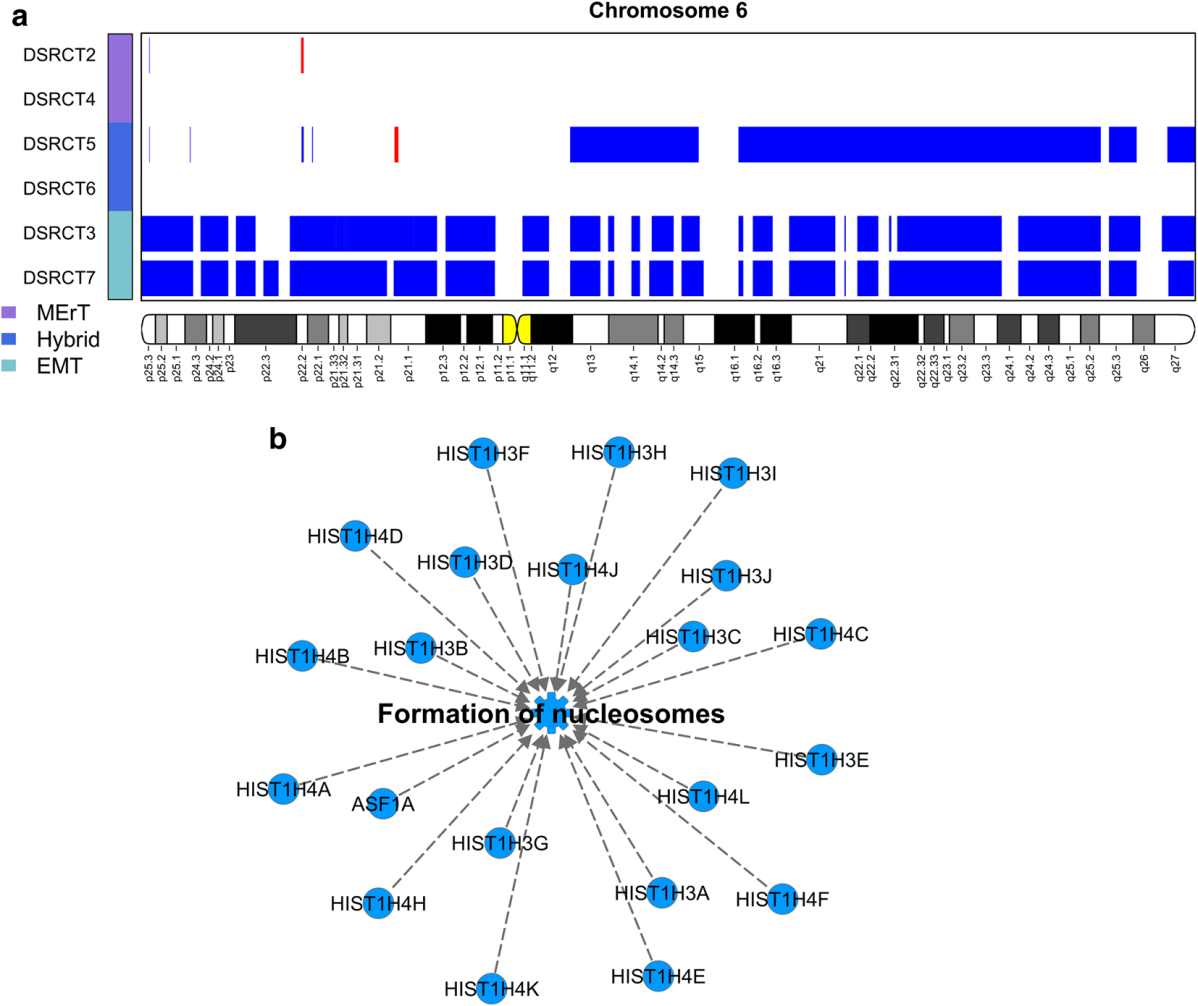
Chromosome 6 losses in DSRCTs. **a** Plot representing genomic location of deleted segments of chromosome 6 in the six DSRCT cases. Loss of the entire chromosome 6 is present in DSRCT3 and 7, but restricted to the long arm in DSRCT5 (see also Additional file 1: Figure S3). The cases are classified into three groups (see coloured vertical bars on the left). **b** Network, identified by Ingenuity Pathway Analysis (IPA^®^, Qiagen; Bioinformatics, Redwood City, CA, USA; http://www.qiagen.com/ingenuity): 21 genes present on the deleted chromosome 6q and belong to the pathway “formation of nucleosome” (total 22 genes) are members of the histone H1 (*HIST1H*) family

A recurrent gain of the long arm of chromosome 1 was identified in four cases (DSRCT2, 3, 5, and 7) (Fig. 6a), loss of the entire chromosome 6 in two cases (DSRCT3 and 7), and loss restricted to the long arm of chromosome 6 in one case (DSRCT5) (Fig. 7a and Additional file 1: Figure S3). Given the high frequency of these CNAs across the six cases, we hypothesized that these regions could contain genes relevant to DSRCT biology, and we performed a functional analysis through IPA.

Regarding chromosome 1, 410 genes were commonly amplified in the four cases (Additional file 2: Table S5). Remarkably, the majority of amplified genes was found to be associated with “Cell movement” (77 amplified genes) and “Cell migration” (68 amplified genes, also present in Cell movement) (both *P *< 0.001) (Fig. 6b and Additional file 2: Table S6). Among these genes, laminin subunit gamma 2 (*LAMC2*), paired related homeobox 1 (*PRRX1*), and regulator of G protein signaling 4 (*RGS4*) are reported to be greatly involved in the dynamic and reversible MErT/EMT process.

Among the 526 genes mapping on chromosome 6q and lost in half of the cases (Additional file 2: Table S7), the category “cellular assembly and organization, DNA replication, recombination, and repair” most significantly enriched in lost genes was involved in the DDR network with 21 gene members of the histone H1 family and related to formation of nucleosomes (*P* < 0.001) (Fig. 7b).

Finally, since the short arm of chromosome 6 carries the major histocompatibility complex (Additional file 2: Table S7) which encodes HLA class I antigens, mandatory for tumor cells to be recognized by cytotoxic T cells, the two monosomic cases (DSRCT3 and 7) are expected to show characteristics of immunoescape [32]. Further strength to a deficient immune response in these two cases is given by the loss of the following genes located on short arm of chromosome 6: proteasome subunit beta 8 (*PSMB8*) and proteasome subunit beta 9 (*PSMB9*), transporter 1, ATP-binding cassette subfamily B member (*TAP1*) transporter 2, ATP-binding cassette subfamily B member (*TAP2*), and TAP-binding protein (*TAPBP*) associated with antigen presentation; and interferon regulatory factor 4 (*IRF4*, also named *MUM1*) recently demonstrated to be fundamental in generation of Type 1 T helper (Th1) response [33].

## Discussion

It has been reported that DSRCT carries a driven translocation, *EWSR1*-*WT1* [34], and is a simple sarcoma with a low frequency of genetic deregulations [4–7]. Our WES and CNA analyses showed that a sizable number of somatic mutations, CNAs, and chromosome imbalances affected genes involved in DDR network, MErT/EMT, and immune response.

Molecular classification of sarcomas splits these tumors into two broad categories: simple sarcomas with near diploid karyotype and simple genetic alterations including translocation or specific activating mutations; complex sarcomas with complex/unbalanced karyotypes [35]. Even if both sarcoma categories show a low somatic mutation burden [36], this is particularly true for simple sarcomas (to which DSRCT belongs) which are thought to be governed by a gene translocation (the EWSR1-WT1 translocation in the case of DSRCT). Consistently, in a comprehensive and integrated genomic characterization of adult sarcomas [36], the 10 cases of synovial sarcoma, the only analyzed type of simple sarcomas, displayed few CNAs and very few mutations with any recurrent one. Interestingly, synovial sarcoma shares many features with DSRCT being characterized by a similar gamut of morphologic/phenotypic changes, namely shifting from epithelial to spindle cell/sarcomatous patterns. Furthermore, a study on malignant pleural mesothelioma, a tumor expressing a refractory profile and carrying EMT features, reported a low mutational burden clustering in DNA repair genes [37].

Although therapy-induced alterations could be expected in our DSRCT cases, being all samples taken after at least two cycles of chemotherapy, the comparison of our WES mutational profile with COSMIC cancer signatures pointed to absence of mutation profiles associated with treatment. Of note, Signature 1 is the result of an endogenous mutational process and is a common signature in most human cancer types, whereas Signature 3 indicates defective homology-directed double-strand DNA break repair and is associated with germline and somatic *BRCA1* and *BRCA2* mutations in breast, pancreatic, and ovarian cancers [38]. Both COSMIC Signatures 1 and 3 were present in sarcomas [36]. Signature 29 has been found in oral cancer samples from individuals with a tobacco chewing habit, but there is evidence that it might be associated with nucleotide excision repair defects.

Overall, considering our WES and CNAs data, we focused our analyses on the DDR network and MErT/EMT pathways, hypothesizing that the co-occurrence of a tumor with refractory characteristics and deregulation of these specific network/pathways may contribute to the distinctive traits of DSRCT.

Given the biological relevance of genes belonging to the DDR network or associated with DDR, including RBPs [39], it has been suggested that their mutations should be added to the list of cancer hallmarks [40] as the eleventh item under the name of “epigenetic and RNA deregulation” [41]. RBPs play a pivotal role in maintaining genome integrity [42] and, acting as a hub, interact with proteins, coding and non-coding RNAs to create a network that regulates crucial steps in the process from the site of DNA damage to pre-mRNAs encoding DDR proteins [43]. As a result of this interplay, RBP alterations are drivers in oncogenesis and lead to a wide range of cellular dysfunctions despite of their low expression levels [28].

After pathway analysis and manual curation of the entire list of identified mutated genes, we observed that 26 of the 135 genes belonged directly/indirectly to the DDR network. Altogether, the observed DDR network-associated mutations affected genes that play a role in various steps of transcription, more frequently the early steps of RNA biogenesis, but also in termination phase, translation, and miRNA production in addition to genes involved in chromatin remodelling. Noteworthy, the two genes involved in the DSRCT driver translocation are components of the DDR network, being the first an RNA binding protein and the second a zinc finger transcription factor.

Manual curation of the entire list of identified mutated genes also indicated that 10 of them belonged to the MErT/EMT pathway in keeping with our data concerning gene expression on the same DSRCT specimens [9]. In fact, we previously suggested that *ZEB1* and miRNA34 (in the form of a miRNA/protein chimera) may drive the shift either towards EMT or MErT according to the prevalence of expression of *ZEB1* over that of miRNA34 or the reverse, respectively [9]. literature data showed that an increase in mesenchymal switching may be achieved by means of the *ZEB1*-induced repression of the RBP epithelial splicing regulatory protein 1 (ESRP1) [44], but a decrease by means of miRNA34-induced repression of the RBP Musashi1 (Ms1) [45]. Thus, the present data may support the observation that RBPs could be key players in miRNA processing [46].

The CNAs’ analysis focused to the DDR network and the MErT/EMT pathway showed that, unlike mutations, most CAN events were recurrent and occurred in genes involved in the MErT/EMT process. Cumulatively, the DSRCT CNA profile points to a deregulation of RNA transport/translation and disturbance of chromatin remodeling, coupled with the amplification of the recently described master factor of hybrid EMT (*ETS2*), along with attenuation of one gene whose deficiency favors EMT (*FOXQ1*) and one gene whose deficiency favors MErT (*FOXF2*), in keeping with the ability of the MErT/EMT process to induce or revert EMT; the identification of an hotspot on the chromosome 21, where in a small area were present the amplified *ETS2*, which promotes EMT, and *HMGN1* and *BRWD1* are involved in epigenetic regulation, a critical component of the MErT/EMT process.

Finally, the identification of loss of short arm of the chromosome 6 in 2 of the six cases, in line with their strong MErT/EMT traits [9], complemented and reinforced the suggestion that DSRCT has a “non-inflammatory tumor type” profile and could be included into the “non-inflammed EMT/stem like” category [47].

Limitations of the present study that should be considered rest essentially in (1) the low number of analyzed cases and (2) the already commented plasticity and time-dependent modulation of the profiles of the three DSRCT groups [9]. Furthermore, we are aware that our focusing only to the DDR network and MErT/EMT pathways may result in a potential underestimation of other altered network/pathways. Further analyses are ongoing in our research group and the public availability of the entire list of mutated genes and CNA losses/gains could enable other researchers to better decipher the entire landscape of DSRCT genomic alterations. Thus, we think that what it remains mandatory is to (1) confirm our new findings in large series of preferably untreated DSRCT cases and (2) functionally prove that the identified genes harbour evidence for a DDR and/or MErT/EMT involvement.

The data described in this paper seem to reveal previously unknown characteristics of DSRCT and, if confirmed, may be useful in elucidating the complex pathogenesis and poor responsiveness to treatment of this type of simple sarcomas as well as in identifying potentially actionable alterations that may allow better tuning of current treatments for this ominous pathology.

## Conclusions

In this study, we showed that, in addition to the *EWSR1*-*WT1* translocation (involving two DDR network proteins), another distinguishing trait of DSRCT could be an aberrant/defective DDR. Furthermore, we reconfirmed the relevance of the MErT/EMT process which, together with the deficient DDR, could lead to the tumor extreme heterogeneity, promote genomic instability, and eventually give rise to drug resistance. Finally, the chromosome 6 loss seems to reconfirm the “immunologically ignorant” signature of this rare and ominous tumor.

## Additional files

**Additional file 1: Figure S1.** Workflow of data processing and bioinformatics pipeline for whole-exome sequencing (WES) and copy number alteration (CNA) analyses. Raw sequence data obtained with Illumina NextSeq500 (Illumina, San Diego, CA, USA) were aligned to the reference genome (hg 19) with Burrows-Wheeler Aligner (BWA) algorithm. Somatic mutations and CNAs were identified using MuTect2 (https://software.broadinstitute.org/gatk/documentation/tooldocs/3.8-0/org_broadinstitute_gatk_tools_walkers_cancer_m2_MuTect2.php) and EXCAVATOR2, respectively. We excluded variants matching at least one of the following criteria: 1) if a variant is supported by 1 or more reads in matched normal sample; 2) if read depth of variant position is < 50, or the variant is supported by less than 10 reads; 3) C>T / G>A variants with a frequency less than 0.1 (possible FFPE artifacts); 4) C>A / G>T variants with a frequency less than 0.1 (possible artifactual mutations due to oxidative DNA damage during sample preparation); 5) if indels are supported only by forward or reverse reads. Biological functions associated with altered genes were investigated through Ingenuity Pathway Analysis (IPA®, Qiagen; Bioinformatics, Redwood City, CA, USA; https://www.qiagen.com/ingenuity). **Figure S2.** Quality control of sequence data analyzed with Conpair. **a)** each tumor-normal pair was correctly matched with a sample concordance above 99%; **b)** levels of cross sample contamination among tumor-normal pairs are negligible. **c)** after removal of duplicates reads, the mean coverage for tumor samples ranged from 109× to 124×, while for normal samples from 52× to 72×. **d)** The percentage of target bases covering at least 50× ranged from 71.4% to 88.8% for tumor samples and from 46.0% to 71.9% for normal samples. **Figure S3.** Copy number profiles for chromosome 6. Profiles of log2 ratio values estimated by EXCAVATOR2 for chromosome 6 of each DSRCT case. Segmented values are represented by the red line.

**Additional file 2.** Table S1. List of somatic mutations identified in each patient. **Table S2.** List, information and literature supply for genes mutated or copy number altered described in the main text as belonging to DDR or MErT/EMT categories. **Table S3.** List of somatic copy number aberrations identified by EXCAVATOR2. **Table S4.** List of gains with at least two copies and losses with homozygous deletions. **Table S5.** List of recurrent amplified genes on chromosome 1. **Table S6.** List of recurrently amplified genes of chromosome 1, belonging to the two significant biological functions identified by Ingenuity Pathway Analysis (IPA®, Qiagen; Bioinformatics, Redwood City, CA, USA; http://www.qiagen.com/ingenuity). For the entire name of the genes, reported as gene ID, see Table S5. **Table S7.** List of recurrent deleted genes on chromosome 6.

## Abbreviations

CNA: copy number alteration
CTA: cancer–testis antigen
DDR: DNA damage-response
DRBP: DNA/RNA binding protein
DDRBPs: DNA damage response RNA binding proteins
DSRCT: desmoplastic small round cell tumor
EMT: epithelial mesenchymal transition
FFPE: formalin-fixed, paraffin-embedded
GATK: Genome Analysis Toolkit
HLA: human leukocyte antigen
IHC: immunohistochemistry
IPA: Ingenuity Pathway Analysis
ISH: in situ hybridisation
MErT: mesenchymal epithelial reverse transition
NGS: next-generation sequencing
PCR: polymerase chain reaction
RBP: RNA binding protein
WES: whole-exome sequencing
