# Supplementary figures and images for: The genomics of desmoplastic small round cell tumor reveals the deregulation of genes related to DNA damage response, epithelial–mesenchymal transition, and immune response
Source: Cancer Commun (Lond). 2018 Nov 28;38:70. doi: 10.1186/s40880-018-0339-3 (PMC6260689; doi:10.1186/s40880-018-0339-3)

## Slide 1
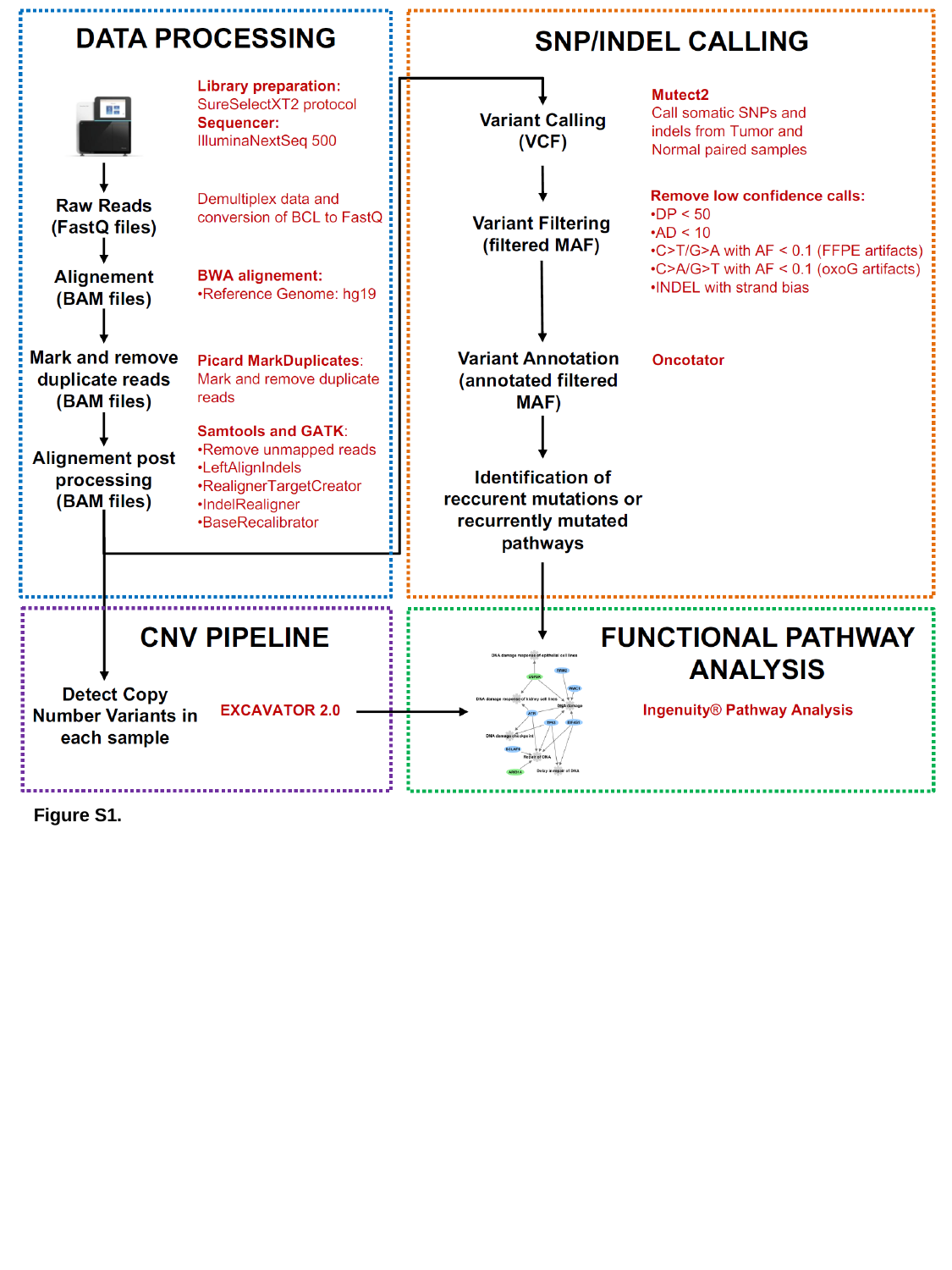

Figure S1.

## Slide 2
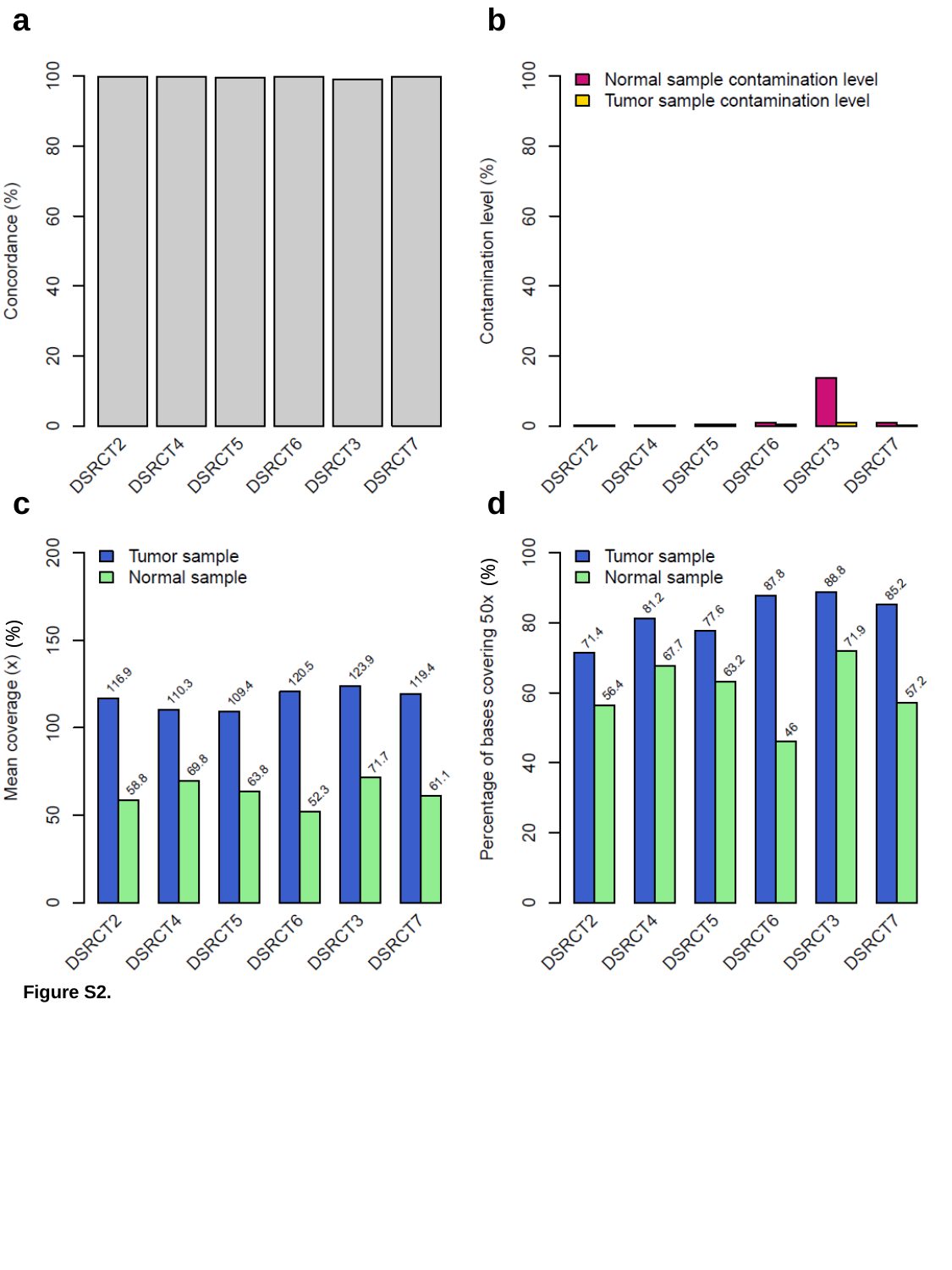

a
b
c
d
(%)
(%)
Figure S2.

## Slide 3
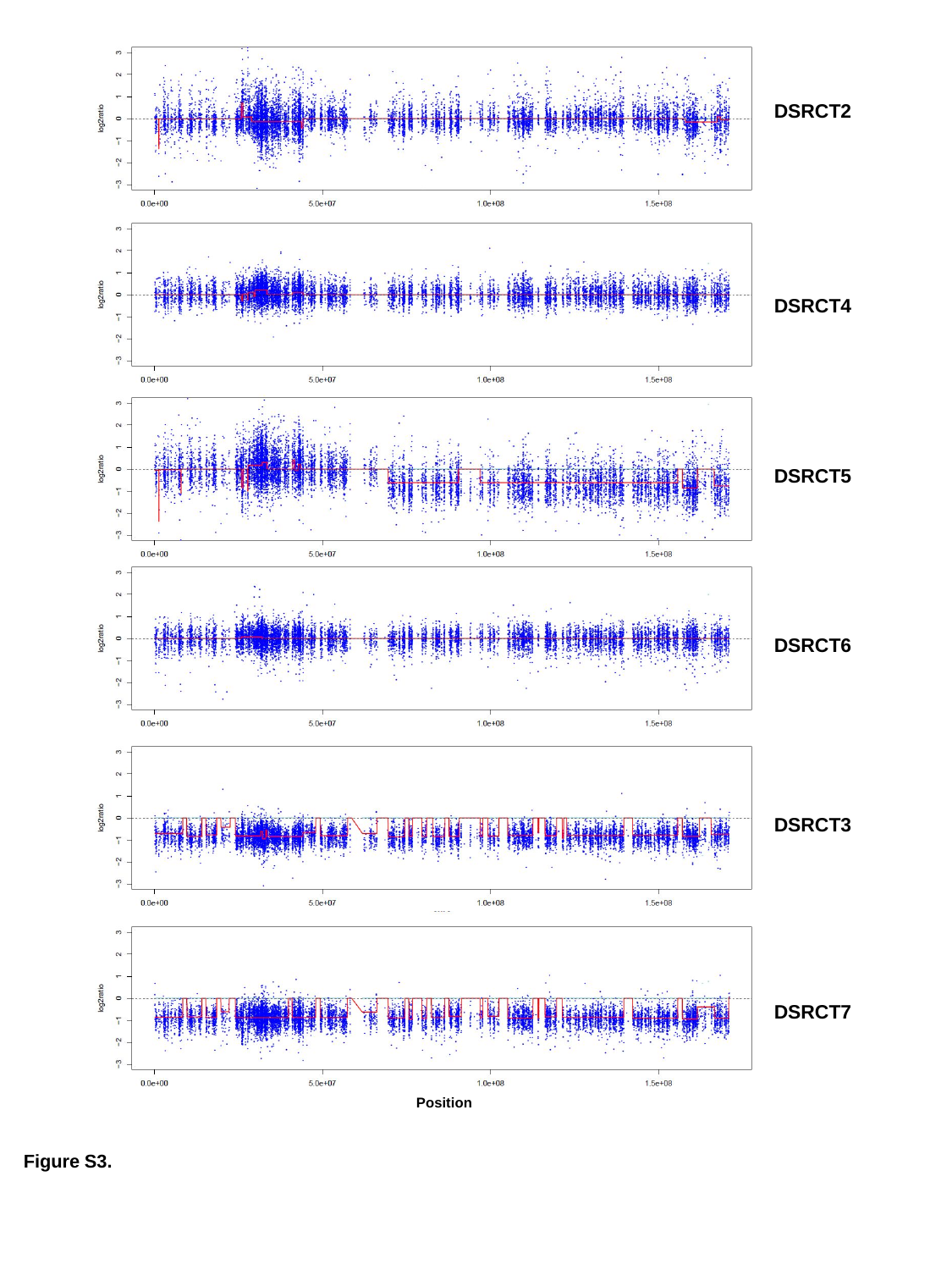

DSRCT2
DSRCT4
DSRCT5
DSRCT6
DSRCT3
DSRCT7
Position
Figure S3.

Supplement: Supplementary file 1 — Additional file 1: Figure S1. Workflow of data processing and bioinformatics pipeline for whole-exome sequencing (WES) and copy number alteration (CNA) analyses. Raw sequence data obtained with Illumina NextSeq500 (Illumina, San Diego, CA, USA) were aligned to the reference genome (hg 19) with Burrows-Wheeler Aligner (BWA) algorithm. Somatic mutations and CNAs were identified using MuTect2 (https://software.broadinstitute.org/gatk/documentation/tooldocs/3.8-0/org_broadinstitute_gatk_tools_walkers_cancer_m2_MuTect2.php) and EXCAVATOR2, respectively. We excluded variants matching at least one of the following criteria: 1) if a variant is supported by 1 or more reads in matched normal sample; 2) if read depth of variant position is < 50, or the variant is supported by less than 10 reads; 3) C>T / G>A variants with a frequency less than 0.1 (possible FFPE artifacts); 4) C>A / G>T variants with a frequency less than 0.1 (possible artifactual mutations due to oxidative DNA damage during sample preparation); 5) if indels are supported only by forward or reverse reads. Biological functions associated with altered genes were investigated through Ingenuity Pathway Analysis (IPA®, Qiagen; Bioinformatics, Redwood City, CA, USA; https://www.qiagen.com/ingenuity). Figure S2. Quality control of sequence data analyzed with Conpair. a) each tumor-normal pair was correctly matched with a sample concordance above 99%; b) levels of cross sample contamination among tumor-normal pairs are negligible. c) after removal of duplicates reads, the mean coverage for tumor samples ranged from 109× to 124×, while for normal samples from 52× to 72×. d) The percentage of target bases covering at least 50× ranged from 71.4% to 88.8% for tumor samples and from 46.0% to 71.9% for normal samples. Figure S3. Copy number profiles for chromosome 6. Profiles of log2 ratio values estimated by EXCAVATOR2 for chromosome 6 of each DSRCT case. Segmented values are represented by the red line. [file 40880_2018_339_MOESM1_ESM.pptx]
